# Secondary Education and Health Outcomes in Young People from the Cape Area Panel Study (CAPS)

**DOI:** 10.1371/journal.pone.0156883

**Published:** 2016-06-09

**Authors:** Joseph L. Ward, Russell M. Viner

**Affiliations:** UCL Institute of Child Health, University College London, London, United Kingdom; National Institute of Health, ITALY

## Abstract

**Aim:**

Education is one of the strongest social determinants of health, yet previous literature has focused on primary education. We examined whether there are additional benefits to completing upper secondary compared to lower secondary education in a middle-income country.

**Methods:**

We performed a longitudinal analysis of the Cape Area Panel Study, a survey of adolescents living in South Africa. We undertook causal modeling using structural marginal models to examine the association between level of education and various health outcomes, using inverse probability weighting to control for sex, age, ethnicity, home language, income, whether employed in past year, region of birth, maternal educational status, marital status, whether currently pregnant and cognitive ability. Educational attainment was defined as primary (grades 1–7), lower secondary (grades 8–9) or upper secondary (grades 10–12).

**Results:**

Of 3,432 participants, 165 (4.8%) had completed primary education, 646 (18.8%) lower secondary and 2,621 (76.3%) upper secondary. Compared to those completing lower secondary, males completing upper secondary education were less likely to have a health problem (OR 0.49; 95%CI 0.27–0.88; p = 0.02); describe their health as poor (0.52; 0.29–0.95; p = 0.03) or report that health interferes with daily life (0.54; 0.29–0.99; p = 0.047). Females were less likely to have been pregnant (0.45; 0.33–0.61; p<0.001) or pregnant under 18 (0.32; 0.22–0.46; p<0.001); and having had sex under 16 was also less likely (males 0.63; 0.44–0.91; p = 0.01; females 0.39; 0.26–0.58; p<0.001). Cigarette smoking was less likely (males 0.52; 0.38–0.70; p = <0.001; females 0.56; 0.41–0.76; p<0.001), as was taking illicit drugs in males (0.6; 0.38–0.96; p = 0.03). No associations were found between education and alcohol use, psychological distress, obesity, increased waist circumference or hypertension.

**Conclusion:**

Completing upper secondary education was associated with improved health outcomes compared with lower secondary education. Expanding upper secondary education offers middle-income countries an effective way of improving adolescent health.

## Introduction

Education has been shown to be one of the strongest social determinants of health.[1] Children who are educated go on to live longer, healthier lives as adults, with less morbidity and disability, with particular benefits seen in young women.[2, 3] This association is seen within countries regardless of their level of development.[4] The influence of education is intergenerational; more educated parents have healthier children, later in life, and fewer of them. In particular maternal level of education has a profound impact on child health outcomes[5]; every additional year of maternal education is associated with a 7%-9% decrease in under 5 child mortality.[6] Half of the reduction in child mortality witnessed globally since 1970 can be attributed to increased education of young women.[2]

There is international consensus regarding the importance of expanding education, and Millennium Development Goal 2 aimed for universal primary education provision.[7] Significant progress has been made[2] and the Institute for Health Metrics and Evaluation (IHME) estimate average completed years of education has increased since the 1990s by 35% for women and 30% for men.[8] Significant challenges remain however, particularly to reduce gender inequities in education. UNESCO estimate there are 15 million children worldwide who will never go to school, of whom 10 million are girls.[9]

The relationship between education and health is complex and varies according to socioeconomic group.[3] Although some explanations are bidirectional[10], there is also evidence of causal pathways of increasing education leading to better health.[4] Firstly, education brings increased earnings, rank and access to resources, which not only independently improve health but also affect the peers that individuals spend time with, which may encourage or discourage healthy behaviours. Education also brings knowledge of adverse health behaviours and better understanding of their implications, and so a greater likelihood of translating beneficial intentions into actions. Education may also bring a greater willingness and understanding of the need to invest in behaviour change now so as to enjoy future health benefits. Finally, those in school are prevented from participating in unhealthy or risky activities during the time in class; the so called “incarceration effect.”[11]

Although there is evidence of a dose response effect of education on health[11], there is less agreement as to he shape of this relationship.[3] Additional years above a certain threshold in adolescents may yield more profound health benefits, and the level for this may vary according to health outcome. Secondary education has been shown to reduce teenage fertility[12] and increase contraception use[13] within low and middle-income countries. Globally, the percentage of immunized children has been shown to be higher when mothers have some secondary education compared with primary or no education.[14] However, the focus of research as to the effects of education on health has concentrated on primary school, with the influence of additional years of education among adolescents less well described outside of high-income countries. We found no previous studies exploring how upper secondary education effects health amongst adolescents compared with lower secondary school within middle-income countries.

South Africa provides a useful opportunity to study the benefits of upper secondary education. Expenditure on education is one of the highest in sub-Saharan Africa, and state funded compulsory education is provided for nine years from age 7 to 15. In 2005 about 85% of South-African primary school aged children, and 65% of secondary school aged children were enrolled in school.[15] Substantial numbers also continue to upper secondary education, providing an opportunity to study the benefits of upper secondary compared with primary / lower secondary education for health.

We used causal modelling methods and data from a longitudinal South African cohort study to explore the benefits of upper secondary education on health outcomes over and above those gained through primary and lower secondary school.

## Methods

We performed a longitudinal analysis using data from the Cape Area Panel Study (CAPS)[16], a survey of young adults aged 14–22 living in Cape Town, South Africa, conducted over 5 waves from 2002 to 2009. Data are publically available and obtained from the University of Cape Town DataFirst portal (https://www.datafirst.uct.ac.za) on 20 August 2015. We primarily used wave 4 of the study for our analysis as this provided the largest sample size, (3,439 participants), with data on multiple health outcomes.

Participants for CAPS were selected using a stratified two-stage sample design. Clusters were selected according to predominant ethnic group using data from the 1996 census (African, white or coloured; a term used in South Africa to describe mixed heritage), with oversampling of white and African clusters to achieve a representative sample. For further details regarding the sampling strategy used in CAPS please see previous methodology.[16]

Ethical approval for CAPS was granted by the University of Cape Town, University of Michigan and University of Princeton. Written consent was obtained from all respondents, and written parental consent for respondents under 18. No ethics approvals were required for the secondary data analyses presented here.

### Education measures

We defined level of education attainment using data collected in wave 4 with the question “What is the highest grade in school that you have successfully completed?” Participants who had received no schooling, or answered “don’t know” or “other” were excluded from the analysis (n = 4).

The primary focus of our analysis was to compare those attending school into the late teens with those leaving education in mid-adolescence. We therefore defined educational attainment as “upper secondary” if participants had completed any years of secondary school beyond grade 9, the limit of compulsory education provided by South Africa and where students are typically 15–16. Those receiving up to the compulsory level of schooling were then divided into “lower secondary”, (high school grades 8–9), or “primary” (grades 1–7). These categories are in line with the International Standard Classification of Education provided by UNESCO.[17]

### Health outcome measures

We used the following questions to identify adverse health outcomes or behaviours among participants, all collected during wave 4:

#### General health

Poor general health was defined using the following questions:

1. “Do you have any health problems or disabilities?”
2. Answering “poor” or “fair” to the question: “In general, how is your health?”
3. Answering “occasionally” “fairly often”, “most of the time”, or “always” to the question: “How often does poor health or physical disability interfere with your ability to study, to work, or to search for work?”

#### Substance use

Participants were asked about any cigarette smoking, alcohol consumption or illicit drug use over the past 30 days.

#### Sexual health

We assessed sexual health amongst participants using the following indicators:

1. Any previous pregnancies.
2. Teenage pregnancy (18 years or younger).
3. Sexual intercourse before the age of 16.

#### Mental health

We defined participants with “moderate” or “severe” scores using the K6 screening scale[18] as having psychological distress.

The K6 screening scale[18] is a 6 item likert scale, with respondents recording how often over the past 30 days they have felt nervous, hopeless, restless, sad, worthless and that everything was an effort. Those with a total score of ≥5 or ≥13 are likely to have “moderate”[19] or “severe”[20, 21] psychological distress respectively.

#### Anthropometry

Obesity; those with a BMI≥30kg/m^2^ were classified as obese.[22]

1. Increased waist circumference; those with a waist circumference above 88cm for females and 102cm for males were classified as having waist circumference above threshold.[23]
2. Hypertension; those with a systolic blood pressure ≥140mmHg or a diastolic blood pressure ≥90mmHg were classified as hypertensive.

### Potential confounding factors

Given that education and health are both likely to be associated with socio-demographic factors, we included the following covariates in our analysis, collected in either wave 1 or wave 4 of the study:

**Variables collected in wave 1**
i. Ethnicity; ethnic group of participants was defined as: “Black/African”, “Coloured”, “Indian”, “White”, “Other”, “Don’t know.”
ii. Language; the language that participants speak most often at home was defined as; “English”, “Xhosa”, “Afrikaans”, “Sotho”, “Zulu”, “Tswana”, “Other”.
iii. Household income; for socioeconomic status we used the log of per capita household income, as others have done.[24, 25]
iv. Region of birth; participants were given the following options: Cape Town, the nine provinces of South Africa, or outside South Africa.
v. Cognition; z-scores from a literacy and numeracy evaluation completed at the start of the study were used as a measure of cognition amongst participants.
vi. Parental educational attainment; defined using total number of years of schooling completed by each participant’s mother and father.

**Variables collected in wave 4**
vii. Age.
viii. Employment; any participants who had been employed in the previous year.
ix. Marital status; this was defined as “Never married”, “Married”, Divorced”, “Separated”, “Widowed”, “Refused”, “Don’t know”.
x. Currently pregnant.
xi. HIV status.

### Statistical Analysis

We initially examined the distribution of each health outcome at wave 4 by education status, using chi square (x^2^) analyses. We then examined the association between education status and health outcome in two ways. First we used standard multivariable logistic regression, including potential covariates in the model and accounting for CAPS survey design using appropriate weighting.[16] Second we repeated these analyses using structural marginal models (SMM) including inverse probability weighting (IPW) to estimate the controlled direct effects of upper secondary education on health outcomes. The use of IPW constructs a pseudopopulation in which the exposure is independent of the factors included in the construction of the weighting. The weighted regression models in the pseudopopulation can then be used to estimate the average causal effect of exposure in the original study population.[26]

Here stabilized IPW were constructed including the following covariates: sex, age, ethnicity, home language, income, whether employed in past year, region of birth, maternal educational status, marital status, whether currently pregnant and cognitive ability. Paternal education level was initially included but dropped due to low sample sizes. Prevalence of HIV was considered for weighting but not included as prevalence within the whole sample was 2%, which appears too low to be representative given a national prevalence amongst 15–24 year olds of 8.7% in 2008[27], and likely reflects under-reporting and/or attrition of HIV-positive people from follow-up. SMM were run as logistic regression models, weighted using the stabilized IPW and including the cluster option for individuals to account for clustering in the longitudinal analysis. All analyses were performed using Stata 14 (StataCorp, College Station TX).

## Results

Of 3,439 participants interviewed in wave 4 of CAPS, data on educational attainment were available for 3,432 (99.8%). Demographic details of the sample and levels of educational attainment are given in Table 1.

**Table 1:** Socio-demographic characteristics by gender amongst participants.

Demographic Variable		
	Males n = 1559	Females n = 1873
	% (n)	% (n)
Mean age at wave 4 (SD)	21.5 (2.70)^a^	21.5 (2.54)^a^
Worked in the past year	63.8 (995)	51.9 (973)
Currently Pregnant	n/a	3.4 (63)
Marital Status		
Never Married	95.6 (1490)	89 (1667)
Married	4.3 (67)	10.4 (194)
Separated/Widowed	0	0.6 (12)
Refused	0.1 (2)	0
Ethnicity		
African	44.7 (697)	47.7 (894)
Coloured	46.9 (731)	45.4 (850)
Indian	0.3 (5)	0.3 (6)
White	8.1 (126)	6.6 (123)
Language at home		
Xhosa	43.3 (673)	46.5 (871)
Afrikaans	37.7 (586)	35.3 (660)
English	18.3 (284)	17.5 (327)
Sotho	0.6 (10)	0.5 (9)
Other	0.1 (2)	0.2 (5)
Location of birth		
Cape Town	71.2 (1110)	67.9 (1271)
Eastern Cape	21.6 (337)	23.2 (435)
Other	7.2 (112)	8.9 (167)
Educational Attainment		
Primary (Grades 1–7)	6.3 (98)	3.6 (67)
Lower Secondary (Grades 8–9)	21.2 (331)	16.8 (315)
Upper Secondary (Grades 10–12)	72.5 (1130)	79.6 (1,491)
Maternal education^b^	38.5 (539)	37.8 (640)

^a^mean age of participants (SD);

^b^ maternal education attainment—completed more than 9 years of education %(n).

Table 2 shows the prevalence of adverse health outcomes within the sample. Table 3 shows the proportion of each health outcome by educational attainment amongst males and females, and adjusted odds ratios for health outcomes, using lower secondary education as the reference group.

**Table 2:** Prevalence of adverse health outcomes by gender amongst participants.

Health Outcomes		
	Males	Females
	%(n)	%(n)
General Health		
Describes health as fair or poor	5 (78)	4.4 (82)
Reports having health problem / disability	4.9 (76)	6.1 (115)
Health interferes with study / work occasionally, fairly often, most of the time	5.5 (85)	7.3 (136)
Substance use		
Smoked cigarettes in the past 30 days	48.8 (760)	24 (452)
Consumed alcohol in past 30 days	44.6 (694)	22.8 (426)
Consumed illicit drugs in past 30 days	8.3 (129)	1.2 (23)
Sexual and reproductive health		
Ever been pregnant / made someone pregnant	20.2 (314)	43.6 (816)
Pregnancy under the age of 18	N/A	13.6 (259)
Had first sexual intercourse under 16	27 (359)	13.2(222)
Mental Health		
Severe / moderate distress on K-6 scale	16.3 (254)	19.2 (359)
Anthropometry		
Obese (BMI >35m^2^)	7.7 (116)	20.1 (368)
Waist above threshold^a^	4.9 (74)	40.3 (735)
Hypertension^b^	17.0 (257)	10.0 (184)

^a^ females >88cm, males > 102cm;

^b^ systolic blood pressure ≥140mmHg or diastolic blood pressure ≥90mmHg)

**Table 3:** Health outcomes by educational attainment for males and females, and adjusted* odds ratios using the logistic regression model, with lower secondary education as the reference group.

		Males			Females	
	% (n)	Odds Ratio* (95% CI)	p	% (n)	Odds Ratio* (95% CI)	p
Poor/ fair general health	n = 1556	n = 1306		n = 1872	n = 1624	
Primary	6.1 (6)	0.81 (0.29–2.28)	0.7	11.9 (8)	1.75 (0.60–5.18)	0.3
Lower Secondary	8.2 (27)	1		7.6 (24)	1	
Upper Secondary	4 (45)	0.53 (0.29–0.97)	0.04	3.3 (50)	0.44 (0.24–0.84)	0.01
x^2^ p value	0.01			<0.001		
Report having health problem / disability	n = 1559	n = 1334		n = 1869	n = 1555	
Primary	8.2 (8)	1.16 (0.46–2.93)	0.7	14.9 (10)	1.44 (0.50–4.17)	0.5
Lower Secondary	7.6 (25)	1		9.3 (29)	1	
Upper Secondary	3.8 (43)	0.44 (0.24–0.84)	0.01	5.1 (76)	0.49 (0.28–0.88)	0.02
x^2^ p value	0.01			<0.001		
Health interferes with work / study	n = 1556	n = 1323		n = 1869	n = 1619	
Primary	8.2 (8)	1.30(0.52–3.28)	0.6	14.9 (10)	1.01 (0.36–2.89)	0.9
Lower Secondary	7.9 (26)	1		9.9 (31)	1	
Upper Secondary	4.5 (51)	0.44 (0.24–0.83)	0.01	6.4 (95)	0.62 (0.37–1.05)	0.08
x^2^ p value	0.03			0.005		
Smoked Cigarettes in the past 30 days	n = 1558	n = 1378		n = 1873	n = 1641	
Primary	64.3 (63)	0.98 (0.57–1.70)	0.9	38.8 (26)	1.26 (0.52–3.06)	0.6
Lower Secondary	59.5 (197)	1		35.9 (113)	1	
Upper Secondary	44.3 (500)	0.44(0.32–0.61)	<0.001	21 (313)	0.39 (0.26–0.59)	<0.001
x^2^ p value	<0.001			<0.001		
Consumed Alcohol in the past 30 days	n = 1556	n = 1374		n = 1869	n = 1643	
Primary	45.9 (45)	1.06 (0.63–1.78)	0.8	26.9 (18)	1.31 (0.63–2.73)	0.5
Lower Secondary	43.6 (144)	1		22.2 (70)	1	
Upper Secondary	44.8 (505)	0.786(0.56–1.04)	0.084	22.7 (338)	0.65 (0.45–0.93)	0.02
x^2^ p value	0.9			0.7		
Taken illicit drugs in the past 30 days	n = 1559	n = 1316		n = 1873	n = 1350	
Primary	21.4 (21)	1.46 (0.74–2.90)	0.3	7.5 (5)	6.77 (1.45–31.65)	0.02
Lower Secondary	11.2 (37)	1		1.3 (4)	1	
Upper Secondary	6.3 (71)	0.57 (0.35–0.93)	0.026	0.9 (23)	0.62 (0.17–2.25)	0.5
x^2^ p value	<0.001			<0.001		
Ever been pregnant / made someone pregnant	n = 1556	n = 1213		n = 1872	n = 1585	
Primary	19.4 (19)	0.62 (0.31–1.23)	0.2	64.2 (43)	0.85 (0.41–1.73)	0.6
Lower Secondary	23.3 (77)	1		60.6 (191)	1	
Upper Secondary	19.3 (218)	0.76 (0.51–1.15)	0.2	39.1 (582)	0.45 (0.32–0.64)	<0.001
x^2^ p value	0.3			<0.001		
Pregnancy under the age of 18				n = 1872	n = 1624	
Primary	n/a	n/a		26.9(18)	0.97 (0.46–2.06)	0.9
Lower Secondary	n/a	n/a		23.5(74)	1	
Upper Secondary	n/a	n/a		8.7(129)	0.44 (0.31–0.66)	<0.001
x^2^ p value				<0.001		
First sexual intercourse under 16	n = 1317	n = 1152		n = 1679	n = 1470	
Primary	41.1 (37)	1.34 (0.74–2.41)	0.3	27.9 (17)	1.15 (0.51–2.58)	0.7
Lower Secondary	32.4 (94)	1		22.1 (62)	1	
Upper Secondary	24.3 (228)	0.73 (0.51–1.05)	0.09	10.7 (142)	0.51 (0.34–0.76)	0.001
x^2^ p value	<0.001			<0.001		
Severe of moderate distress on K-6 Scale	n = 1559	n = 1365		n = 1873	n = 1643	
Primary	23.5 (23)	1.55 (0.82–2.94)	0.2	26.9 (20)	1.74 (0.85–3.53)	0.1
Lower Secondary	16.6 (55)	1		21.6 (68)	1	
Upper Secondary	15.6 (176)	0.92 (0.62–1.37)	0.7	18.2 (271)	0.80 (0.55–1.14)	0.2
x^2^ p value	0.1			0.029		
Obese (BMI>30m^2^)	n = 1102	n = 1294		n = 1834	n = 1623	
Primary	7.7 (7)	1.65 (0.64–4.28)	0.3	25 (16)	0.99 (0.44–2.26)	0.9
Lower Secondary	5.3 (17)	1		18.4 (57)	1	
Upper Secondary	8.4 (92)	1.27 (0.70–2.33)	0.4	20.2 (295)	1.09 (0.75–1.58)	0.7
x^2^ p value	0.2			0.5		
Waist above threshold	n = 1511	n = 1312		n = 1825	n = 1615	
Primary	4.49 (4)	1.85 (0.51–6.73)	0.4	34.4 (22)	0.55 (0.27–1.12)	0.1
Lower Secondary	3.1 (10)	1		39.1 (120)	1	
Upper Secondary	5.5 (60)	1.23 (0.53–2.86)	0.6	40.8 (593)	0.97 (0.72–1.32)	0.9
x^2^ p value	0.2			0.5		
Hypertension	n = 1517	n = 1339		n = 1842	n = 1617	
Primary	14.3 (13)	1.00 (0.46–2.21)	0.9	10.6 (7)	1.21 (0.42–3.46)	0.6
Lower Secondary	15.5 (50)	1		7.7 (24)	1	
Upper Secondary	17.6 (194)	1.33 (0.86–2.06)	0.2	10.5 (153)	1.13 (0.67–1.90)	0.8
x^2^ p value	0.5			0.3		

*Odds ratios adjusted for age, ethnic group, language spoken at home, per capita income(log), employment in the past year, region of birth, years of maternal education completed, marital status, whether currently pregnant and standardised numeracy and literacy score.

The distribution of the following adverse health outcomes varied significantly according to differing level of educational attainment amongst males and females, using chi squared (x^2^) statistic: poor general health; having a health problem or disability; frequency that health interferes with work or study; smoking and illicit drug use and sex under 16. Having any previous pregnancies, pregnancy under the age of 19 and reporting psychological distress, also varied significantly by educational attainment for females.

When adjusted for hypothesized confounders described above using logistic regression, upper secondary education appeared to be protective for a number of adverse health outcomes, with males and females reporting improved general health and less disability or chronic illness. Males also experienced less interference of health on study or work, but not females. Substance use was also less common; males and females were less likely to have smoked in the past 30 days, females were less likely to have drunk alcohol, and males were less likely to have taken illicit drugs. Reproductive and sexual health was also better amongst those receiving upper secondary education. Females were less likely to have had sex under 16, to have ever been pregnant, to have become pregnant under 19,

When comparing those who had completed primary education with those who had completed lower secondary education, females who were less well educated were more likely to have taken illicit drugs in the past 30 days. No association was found with any of the other health outcomes measured.

Table 4 shows odd ratios from structural marginal models for each health outcome, using inverse probability weighting with the covariates described above, using lower secondary as the reference group. We found similar results to our logistic regression output with participants completing upper secondary education reporting improved health on a variety of indicators, particularly amongst males. Upper secondary education was protective against poor general health, having a chronic health problem, and health interfering with work or study amongst males, but not females. Upper secondary education continued to be protective of adverse sexual and reproductive health amongst females in the sample however, who were less likely to have been pregnant, had a teenage pregnancy or had sex under 16. Males were also less likely to have had sex under 16. Smoking cigarettes was also less common amongst the better-educated participants, as was taking illicit drugs amongst males but not females.

**Table 4:** Health outcomes by educational attainment, structural marginal model odds ratios using inverse probability weighting. (IPW)*

	Males		Females	
	Structural Marginal Models OR (95% CI)	p	Structural Marginal Models OR (95% CI)	p
Poor/ fair general health	n = 1377		n = 1662	
Primary	0.66 (0.15–2.9)	0.6	1.50 (0.33–6.88)	0.6
Lower Secondary	1		1	
Upper Secondary	0.52 (0.29–0.95)	0.03	0.67 (0.37–1.21)	0.18
Report having health problem / disability	n = 1380		n = 1660	
Primary	0.35 (0.10–1.197)	0.09	0.71 (0.14–3.60)	0.7
Lower Secondary	1		1	
Upper Secondary	0.49 (0.27–0.88)	0.02	0.58 (0.33–1.04)	0.07
Health interferes with work / study	n = 1377		n = 1660	
Primary	0.40 (0.13–1.23)	0.11	0.53 (0.12–2.38)	0.4
Lower Secondary	1		1	
Upper Secondary	0.54 (0.29–0.99)	0.047	0.71 (0.42–1.21)	0.2
Smoked Cigarettes in the past 30 days	n = 1379		n = 1663	
Primary	1.44 (0.53–3.88)	0.5	2.4 (0.51–11.36)	0.3
Lower Secondary	1		1	
Upper Secondary	0.52 (0.38–0.70)	<0.001	0.56 (0.41–0.76)	<0.001
Consumed Alcohol in the past 30 days	n = 1377		n = 1659	
Primary	1.08 (0.40–2.90)	0.9	0.31 (0.08–1.19)	0.09
Lower Secondary	1		1	
Upper Secondary	1.01 (0.75–1.37)	0.9	0.89 (0.64–1.27)	0.5
Taken illicit drugs in the past 30 days	n = 1380		n = 1663	
Primary	0.75 (0.31–1.81)	0.5	1.20 (0.16–8.92)	0.9
Lower Secondary	1		1	
Upper Secondary	0.60 (0.38–0.96)	0.03	0.82 (0.26–2.63)	0.7
Ever been pregnant / made someone pregnant	n = 1377		n = 1662	
Primary	0.58 (0.22–1.57)	0.3	0.67 (0.12–3.54)	0.6
Lower Secondary	1		1	
Upper Secondary	0.85 (0.60–1.20)	0.3	0.45 (0.33–0.61)	<0.001
Pregnancy under the age of 18			n = 1662	
Primary	n/a	n/a	0.22(0.06–0.76)	0.02
Lower Secondary	n/a	n/a	1	
Upper Secondary	n/a	n/a	0.32 (0.22–0.46)	<0.001
First sexual intercourse under 16	n = 1161		n = 1489	
Primary	0.68 (0.27–1.69)	0.4	0.36 (0.09–1.43)	0.1
Lower Secondary	1		1	
Upper Secondary	0.63 (0.44–0.91)	0.01	0.39 (0.26–0.58)	<0.001
Severe or moderate distress on K6	n = 1380		n = 1663	
Primary	0.694 (0.27–1.79)	0.5	0.57 (0.16–2.01)	0.4
Lower Secondary	1		1	
Upper Secondary	0.94 (0.62–1.41)	0.7	0.71 (0.48–1.04)	0.08
Obese (BMI>35m^2^)	n = 1343		n = 1633	
Primary	2.85 (0.51–16.02)	0.2	0.50 (0.11–2.21)	0.4
Lower Secondary	1		1	
Upper Secondary	1.39 (0.77–2.52)	0.3	0.89 (0.60–1.31)	0.5
Increased waist for sex	n = 1336		n = 1623	
Primary	0.48 (0.12–1.85)	0.3	0.26 (0.07–1.02)	0.053
Lower Secondary	1		1	
Upper Secondary	1.77 (0.80–3.93)	0.16	0.87 (0.64–1.18)	0.4
Hypertension	n = 1341		n = 1639	
Primary	0.55 (0.20–1.53)	0.2	0.24 (0.06–1.07)	0.061
Lower Secondary	1		1	
Upper Secondary	1.26 (0.79–2.02)	0.3	1.27 (0.74–2.19)	0.4

IPW*: sex, age, ethnicity, home language, income, whether employed in past year, region of birth, maternal educational status, marital status, whether currently pregnant and cognitive ability.

When comparing those who had only completed primary education with those completing lower secondary education using structural marginal models, becoming pregnant as a teenager was less likely within those who had only completed primary education. This contrasts to the logistic regression output, where lower secondary education appeared protective, and we feel can be explained through the low sample size of this outcome within primary school educated women. No other health outcomes were found to be significant when comparing primary and lower secondary educational attainment.

Obesity, hypertension, high waist circumference and psychological distress were not significantly associated with educational attainment amongst males or females using either the logistic regression or structural marginal models.

## Discussion

We found consistent evidence within this longitudinal cohort that continuing education beyond lower secondary school improves a variety of health outcomes for young people. This is the first systematic study of the influence of upper secondary education on broad health outcomes in low and middle-income countries. We found that for young women, upper secondary education was particularly protective against sexual health outcomes, with those continuing to upper secondary being 40 to 60% less likely to be have been pregnant, particularly pregnant < 18 years, or started sex before 16 years compared with those who did not. For young men, upper secondary was more broadly protective across general health, substance use and sexual health. These findings were consistent across traditional longitudinal regression and causal modelling analyses, indicating that the effects of education shown here were independent of sex, age, ethnicity, household income, employment, region of birth, language spoken at home, cognition, and level of maternal education. We did not find evidence of protection of upper secondary education on psychological function or cardiometabolic risk factors.

### Comparison with the literature

Our findings of a reduction in adolescent fertility, early sexual debut and teenage pregnancy among secondary educated females are consistent with other studies in similar settings within sub Saharan Africa. Mahy[12] and colleagues found secondary education to exert a greater protective effect than primary school on early marriage, early sexual debut and teenage pregnancy. Bongaarts[13] and colleagues showed that teenage fertility and desired family size decreased, and contraception use increased, amongst secondary school educated females compared with those who were primary educated only. They suggest education provides greater autonomy within sexual relationships and better knowledge of sexual risk and how to reduce it.[28]

Studies from high-income countries have reported a particular beneficial effect of upper secondary education with regard to self reported health amongst females.[3] In contrast, we found general health to be improved in males but in females this association was significant in the adjusted regression models but not the SMM.

We found upper secondary education to be protective of any cigarette smoking for males and females, which is consistent with other studies in comparable countries. Using data from the World Health Survey comparing smoking rates amongst those primary, secondary and tertiary educated, Hosseinpoor and colleagues[29] found a steep protective gradient as education increased in low and middle-income countries. Completing nine or more years of education was also associated with reduced risk of smoking in one study in Brazil.[30]

We also found better educated males to be less likely to have taken illicit drugs, which is consistent with previous studies undertaken in high-income countries[31, 32], where the vast majority of research in the area takes place despite the substantial burden of substance use in low and middle-income countries.[33] We were unable to find evidence to support this association in comparable settings, and this should be a focus of future research.

We found no association between educational attainment and cardiometabolic risk factors including obesity, high waist circumference and hypertension. This may reflect relatively low prevalence of risk factors in young adults. The only other studies to have examined this association have been in high income countries [34] and shown only modest associations.[3]

In contrast to studies in high-income countries[3] we found no association with secondary education and mental health or alcohol use. We used a score of 5 or more on the K-6 scale to denote moderate psychological distress.[19] A score of 13 has been more widely validated to denote severe distress [20, 21] but using this threshold we would only pick up psychological distress in 2.7% of our sample. Using the full 10-point Kessler scale, or a more detailed assessment of mental health, may have better identified participants with mental health difficulties, and given more confidence in interpreting our findings. Our analysis may have also lacked specificity in identifying alcohol problems among participants, as we included any consumption in the past 30 days as an outcome, which will include both minimal and problem drinking. No information was collected with regard to alcohol misuse, dependency, or age at which participants started drinking.

### Limitations

We used longitudinal data from a population-based South African cohort with high retention from early adolescence to young adulthood. Missing data for our exposure variable, education, were minimal. We used both adjusted logistic regression and SMM causal modelling techniques, the latter ensuring that our estimates of the effects of upper secondary education were independent of a very wide range of potential confounders. We studied a wide range of health outcomes and undertook analyses separately by sex.

Our data were subject to a number of limitations. The outcomes we were able to study were limited by data collected in the surveys, leading to limitations in mental health and substance misuse data as noted above. They also predominantly relied on participants’ subjective interpretation of their health and well-being and may be subject to error. Whilst we used causal modelling techniques, which ensured our findings were independent of all included confounders, we cannot exclude unmeasured confounding nor that an unmeasured common factor was associated with both education and health.

When considering the applicability of our results to other middle-income countries, it is also important to recognise that CAPS was conceived during a period of rapid social, political and economic change in South Africa following the end of apartheid. Although other middle and low-income countries have recently experienced considerable upheaval, those of South Africa are likely to be unique and this should be acknowledged when interpreting our results.

## Conclusion

Our findings are strongly suggestive that higher levels of education provide health benefits additional to those clearly established for lower (e.g. primary) education levels. Whilst causality cannot be assumed, these data add to arguments for countries to extend education to include upper secondary education. The United Nations Sustainable Development Goals include the aim to provide free secondary education for all and tertiary education that is affordable by 2030.[35] This study adds to the growing body of evidence that to do so will improve health outcomes and behaviours within middle-income countries
